# Predicted heart age profile across 41 countries: A cross-sectional study of nationally representative surveys in six world regions

**DOI:** 10.1016/j.eclinm.2022.101688

**Published:** 2022-10-01

**Authors:** Wilmer Cristobal Guzman-Vilca, Gustavo A. Quispe-Villegas, Rodrigo M. Carrillo-Larco

**Affiliations:** a School of Medicine “Alberto Hurtado”, Universidad Peruana Cayetano Heredia, Lima, Peru; b CRONICAS Centre of Excellence in Chronic Diseases, Universidad Peruana Cayetano Heredia, Lima, Peru; c Sociedad Científica de Estudiantes de Medicina Cayetano Heredia (SOCEMCH), Universidad Peruana Cayetano Heredia, Lima, Peru; d Department of Epidemiology and Biostatistics, School of Public Health, Imperial College London, London, UK; e Universidad Continental, Lima, Peru

**Keywords:** Risk assessment, Developing countries, Cardiovascular diseases, Adults

## Abstract

**Background:**

Predicted heart age (PHA) can simplify communicating the absolute cardiovascular disease (CVD) risk. Few studies have characterized PHA across multiple populations, and none has described whether people with excess PHA are eligible for preventive treatment for CVD.

**Methods:**

Pooled analysis of 41 World Health Organization (WHO) STEPS surveys conducted in 41 countries in six world regions between 2013 and 2019. PHA was calculated as per the non-laboratory Framingham risk score in adults without history of CVD. We described the differences between chronological age and PHA, the distribution of PHA, and the proportion of people with excess PHA that were eligible for antihypertensive and lipid-lowering treatment following the WHO guidelines. Logistic regression models were fitted to assess sociodemographic and health-related variables associated with PHA excess.

**Findings:**

94,655 individuals aged 30–74 years were included. 36% of those aged 30–34 years had a PHA of 30–34 years; 9% of those aged 60-64 years had a PHA of 60-64 years. Countries in Africa had the lowest prevalence of very high PHA (i.e., PHA exceeding chronological age in ≥5 years) and countries in Western Pacific had the highest. ≥50% of the population with PHA excess (i.e., PHA exceeding chronological age in ≥1 year) was not eligible for antihypertensive nor lipid-lowering treatment. Abdominal obesity, high total cholesterol, smoking and having diabetes were associated with higher odds of having PHA excess, whereas higher education and employment were inversely associated with excess PHA.

**Interpretation:**

PHA is generally higher than chronological age in LMICs and there are regional disparities. Most people with excess PHA would not be eligible to receive preventive medication.

**Funding:**

RMC-L is supported by a Wellcome Trust International Training Fellowship (214185/Z/18/Z).

**Research in context**

**Evidence before this study**

We searched PubMed on May 17^th^, 2021, with no language or date restrictions. The search strategy was: (“heart age” OR “vascular age”) AND (“prevalence” OR “distribution”) AND (“low- and middle-income countries” OR “developing countries”). The search yielded only one work that described predicted heart age distribution across six low- and middle-income countries. No global work has provided PHA estimates for multiple LMICs. Furthermore, the proportion of people with excess PHA that would be eligible for preventive treatment for cardiovascular diseases (CVD) remains unknown.

**Added value of this study**

We analysed individual-level data from 41 World Health Organizations (WHO) STEPS surveys to provide the largest assessment of PHA across multiple countries (38 LMICs). This study provides important additions to the existing literature. First, although regional disparities exist, PHA is substantially higher than chronological age in LMICs. Second, we have highlighted strong inequities in the global distribution of PHA, characterized by higher prevalence of excess PHA in Western Pacific and high-income countries, and lower prevalence of excess PHA in Africa and low-income countries. Third, most people with excess PHA would not be eligible for antihypertensive nor lipid-lowering treatment following WHO guidelines. The latter is important to inform clinical guidelines, as treating all people with excess PHA could result in overtreating low-risk individuals.

**Implications of all the available evidence**

Despite PHA has emerged as a useful tool to simplify CVD risk communication, evidence about its distribution at the population-level is limited. Our work could spark interest from regional or local organizations to ascertain what countries would benefit the most out of using PHA on clinical practice guidelines of cardiovascular risk assessment.

Alt-text: Unlabelled box

## Introduction

Cardiovascular diseases (CVD) are the main cause of mortality globally,^1^ accounting for approximately 18.5 million deaths and 385.9 million disability-adjusted life years (DALYs) in adults in 2019.^2^^,^^3^ Although global estimates indicate that age-standardised CVD death rates have decreased in the last three decades,^2^^,^^3^ such reduction in low- and middle-income countries (LMICs) has not been as pronounced as in high-income countries.^2^^,^^3^ Therefore, to further reduce the mortality burden of CVD in LMICs as supported by global organizations (e.g., United Nations^4^ and the World Health Organization (WHO)^5^), screening for cardiometabolic risk factors and providing treatment for people at high cardiovascular risk should be a priority.^1^

Calculators have been created to predict the absolute CVD risk over short (e.g., 5 years)- or long-term (e.g., 30 years) periods.^6^, ^7^, ^8^, ^9^ These calculators are now a crucial component of the risk-based approach to prevent CVD in several clinical guidelines.^10^, ^11^, ^12^, ^13^ Nonetheless, communicating the absolute CVD risk can be challenging for physicians and this concept can be difficult to digest by patients.^14^ Simple concepts like predicted heart age (PHA) have emerged as potentially useful tools to simplify communicating the absolute CVD risk.^15^ An individual's PHA represents the chronological age of someone with similar absolute CVD risk but with normal risk factors.^7^ That is, if modifiable risk factors are elevated, the individual's PHA would be higher than his/her chronological age. Because middle-aged adults generally have low absolute cardiovascular risk due to their young chronological age, cardiovascular risk expressed as PHA could be more persuasive to motivate lifestyle changes.^16^ Recent evidence indicates that PHA has the potential to reduce risk factor levels when combined with other techniques (e.g., counseling) to communicate CVD risk.^15^ Nevertheless, few studies have characterized PHA across multiple populations,^17^^,^^18^ and only one focused on six LMICs.^19^ In addition, PHA has been proposed as a tool to inform the need for medication to prevent CVD in clinical guidelines.^20^ Nevertheless, there is controversy on whether PHA can be used to guide preventive treatment for CVD,^16^ as medicating all people with any excess of PHA (regardless of their absolute risk) could result in overtreating people at low CVD risk.

Using nationally representative surveys in 41 countries, we described the distribution of PHA in the general population, along with the proportion of people with excess PHA that is eligible for antihypertensive and lipid-lowering treatment following international guidelines.

## Methods

### Study design

Cross sectional analysis of pooled, individual-level data of nationally representative surveys. This analysis adheres to the Strengthening the Reporting of Observational Studies in Epidemiology (STROBE) guideline. The STROBE Checklist is shown in the Supplementary material.

### Data sources and study population

We analyzed WHO STEPwise Approach to NCD Risk Factor Surveillance (STEPS) surveys. These surveys collect health variables via questionnaires, physical measurements (e.g., blood pressure (BP)), and blood biomarkers (e.g., fasting plasma glucose (FPG)). All of these assessments follow standardized methods.^21^ We sought surveys that fulfilled the following inclusion criteria: 1) nationally representative (i.e., subnational surveys were not included); and 2) most recent survey per country (i.e., only one survey per country).

We performed a complete-case analysis in non-pregnant adults aged 30-74 years without history of CVD; this is the population of interest of the Framingham risk score.^22^ We dropped participants with implausible values of systolic BP (SBP) (outside the range of 70-270 mmHg), diastolic blood pressure (DBP) (outside the range of 30-150 mmHg), height (outside the range of 1.00-2.50 meters), weight (outside the range of 12-300 kilograms), body mass index (BMI) (outside the range of 10-80 kg/m^2^), waist circumference (outside the range of 30-200 centimetres), FPG (outside the range of 45-540 mg/dl), and total cholesterol (outside the range of 67-773 mg/dl). A flowchart of the data cleaning process is presented in Supplementary Figure 1. The number of observations per country at each step of the data cleaning process is presented in the Supplementary Table 1.

### Variables

#### Original variables

As the laboratory Framingham risk score needs high-density lipoprotein (HDL) levels and not all (28 out of 41) surveys measured HDL, we calculated PHA as per the non-laboratory Framingham risk score.^7^ Of note, the c-statistic of the Framingham non-laboratory risk model (0.75 in men and 0.79 in women) is overall similar to the Framingham laboratory risk model (0.76 in men and 0.79 in women) in both sexes.^7^ We performed a sensitivity analysis (shown in Supplementary Table 2) in which PHA was computed using the Framingham laboratory-based risk score (which included HDL cholesterol). To do this, we used data from 66,018 people from 28 surveys (i.e., 28 countries). We then compared CVD risk and PHA (as numeric values computed as per the lab-based score vs the non-lab-based score using paired t-tests).

We selected the Framingham risk score^7^ to be consistent with previous research, as previous studies of the clinical usefulness^15^ and distribution of PHA across multiple countries (including LMICs)^18^^,^^19^^,^^23^^,^^24^ have used the Framingham risk score. We used the following variables to compute PHA in people without history of CVD^7^: sex, age (years), SBP, BMI, treatment for hypertension, smoking and diabetes status. We also included the following variables in multilevel regression models: level of education, marital status, work status, total cholesterol, and abdominal obesity.

CVD history was assessed with the following question: *Have you ever had a heart attack or chest pain from heart disease (angina) or a stroke (cerebrovascular accident or incident)?* As shown in the flowchart of data cleaning (Supplementary Figure 1), only those who responded *no* to this question were included in the analysis.

STEPS surveys collect three blood pressure records along with anthropometric measurements; these are collected by trained fieldworkers following a standard protocol (Supplementary Table 3).^21^ Weight (in kilograms) and height (in meters) were used to calculate BMI (weight divided by the square of height). As the Framingham CVD risk calculator only accepts BMI levels of 15-50 kg/m^2^, BMI values below 15 kg/m^2^ and above 50 kg/m^2^ (0.7% of the pooled dataset) were rounded to 15 and 50, respectively. Regarding SBP, we used the mean of the second and third measurement (i.e., the first measurement of SBP was discarded). The risk calculator only accepts SBP levels of 90-200 kg mmHg; in consequence, as suggested by the Framingham Heart Study,^22^ values below 90 mmHg and above 200 mmHg (1.1% of the pooled dataset) were rounded to 90 and 200, respectively.

Self-reported treatment of hypertension was assessed using the following question (or an equivalent): *In the past two weeks, have you taken any drugs for raised blood pressure prescribed by a doctor or other health worker?* Those who answered *yes* to this question were considered as treated for hypertension.

Smokers were defined based on the following yes/no question: *Do you currently smoke any tobacco products, such as cigarettes, cigars, or pipes?*

Diabetes status was assessed using FPG and self-reported information on diabetes diagnosis: *Have you ever been told by a doctor or other health worker that you have raised blood sugar or diabetes?* We considered people with diabetes as those who had FPG ≥ 126 mg/dl or answered *yes* to the self-reported diagnosis question. The protocol for the measurement of FPG by survey is detailed in the Supplementary Table 3.

Education was ascertained using the following question: *What is the highest level of education you have completed?* Answers for most surveys were originally coded in seven categories: no formal schooling, less than primary school, primary school completed, secondary school completed, high school completed, college/university completed, and postgraduate degree. We grouped these into four categories: none (no formal schooling), some primary/primary (less than primary school and primary school completed), secondary/high (secondary school completed and high school completed), and university or higher (college/university completed and postgraduate degree).

Marital status was assessed using the following question: *What is your marital status?* Answers for most surveys were originally coded in six categories: never married, currently married, separated, divorced, widowed, and cohabitating. We grouped these into three categories: never married, currently married/cohabitating, and divorced/separated/widowed.

Work status was assessed using the following question: *Which of the following best describes your main work status over the past 12 months?* Answers for most surveys were originally coded in nine categories: government employee, non-government employee, self-employed, non-paid, student, homemaker, retired, unemployed but able to work, and unemployed und unable to work. We grouped these into five categories: currently employed and paid (government employee, non-government employee, self-employed), currently employed but not paid (non-paid), student, homemaker, and currently unemployed (retired and unemployed able/unable to work).

Total cholesterol levels were used to assess which individuals had high (≥200 mg/dl) or normal (<200 mg/dl) total cholesterol. The protocol for the measurement of total cholesterol by survey is detailed in the Supplementary Table 1. Abdominal obesity was assessed using waist circumference, which was measured by trained fieldworkers. We classified individuals into two groups: abdominal obesity (waist circumference ≥102 cm in men and ≥88 cm in women) and no abdominal obesity (waist circumference <102 cm in men and <88 cm in women).^25^

#### Derived variables

We calculated PHA based on the non-laboratory Framingham risk score. As only few STEPS surveys measured HDL-cholesterol,^26^ it was not possible to calculate PHA as per the laboratory Framingham risk score. The complex algorithm to compute CVD risk and PHA as per the Framingham risk score is presented in the Supplementary Figure 2 and has been published in depth elsewhere.^7^ First, the 10-year risk of CVD (coronary death, myocardial infarction, coronary insufficiency, angina, ischemic stroke, hemorrhagic stroke, transient ischemic attack, peripheral artery disease, heart failure) is predicted. Second, this risk is compared with the age of someone with the same predicted risk but with all cardiovascular risk factors at their ideal levels: SBP of 125 mmHg, BMI of 22.5 kg/m^2^, non-smoker, not with diabetes nor antihypertensive treatment.^7^^,^^18^ We used the *frisk* R package to compute absolute cardiovascular risk and PHA predictions; this package produces numerical results of PHA except when it is younger than 30 years (coded as “<30”) and higher than 80 years (coded as “>80”).

PHA was then categorized into four levels based on its difference with chronological age: low (i.e., PHA is lower than chronological age), equal (i.e., PHA is equal to chronological age), high (i.e., PHA exceeds chronological age by 1-4 years), and very high PHA (i.e., PHA exceeds chronological age by 5 years or more). The latter two PHA categories were also grouped as excess PHA (i.e., PHA exceeds chronological age).

Among those with excess PHA, we described the proportion of people eligible for antihypertensive and lipid-lowering treatment following the WHO HEARTS technical package.^10^ Although the risk-based approach recommended in these guidelines could be used with other risk calculators,^10^ it would have been ideal if our estimates of absolute CVD risk and PHA followed the 2019 WHO risk charts^6^ because the Framingham risk score has not been recalibrated for most LMICs. Therefore, results should be interpreted considering this limitation. According to the WHO guidelines, the following groups were eligible for antihypertensive treatment: 1) CVD risk between 10%–19% and SBP≥140 or DBP≥90 mmHg; 2) CVD risk ≥20% and SBP≥130 or DBP≥80 mmHg; and 3) all individuals with SBP≥160 or DBP≥100 mmHg.^10^ Those who did not meet these criteria were considered not eligible for antihypertensive treatment. According to the WHO guidelines, the following groups were eligible for lipid-lowering treatment: 1) CVD risk ≥20%, and 2) all individuals ≥40 years with diabetes. Those who did not meet these criteria were considered not eligible for lipid-lowering treatment.

### Statistical analysis

The statistical analyses were conduct with R (version 4.1.2). The analysis code is available as Supplementary material. Absolute numbers, means and proportions were used to describe the characteristics of each survey's sample.

### Differences between chronological and predicted heart age

First, we described the divergence of chronological age across PHA estimates. Because our aim was to observe divergence rather than reporting prevalence estimates at the national level, this analysis did not account for the complex survey design of each survey. Both chronological age and PHA were categorized into 5-year age groups (except for ≤30 and ≥80 PHA groups). Then, sex-specific Sankey plots were used to depict chronological age groups against PHA groups in the overall sample and by world region.

### Prevalence of predicted heart age categories

As not all PHA values were continuous (i.e., PHA of <30 and >80 years), the gap between chronological age and PHA was not reported as a continuous variable. Rather, we categorized this gap into categories of PHA. We described the sex-specific distribution of each PHA category (low, equal, high, and very high). These prevalence estimates accounted for the complex survey design of each survey. For this, we used the *svy* package on R, where the survey design parameters (i.e., primary sampling unit, stratum, sampling weight) were specified for each survey. In addition, boxplots were used to describe these results by world region (according to the WHO classification^27^) and national income (according to the World Bank classification^28^). Differences in prevalence estimates by region and income were assessed with one-way ANOVA tests. Only one country (i.e., Tokelau) was excluded from this analysis by income because they did not have income data.

### Eligibility for antihypertensive and lipid-lowering treatment

We described the proportion of the population with PHA excess that were eligible for antihypertensive treatment and lipid-lowering treatment. Eligibility for treatment was the numerator and PHA excess was the denominator. These estimates accounted for the complex survey design of each survey.

### Potential correlates of excess predicted heart age and eligibility for treatment

We run mixed effects logistic regression models to assess which socio-demographic (level of education, marital and work status) and health-related (abdominal obesity and high cholesterol) variables were linked to having PHA excess. We included random intercepts whereby countries were nested within world regions. The regression results are presented as odds ratios (ORs) along with 95% confidence intervals (95% CIs). We considered *p* values <0.05 as statistically significant. These regression models did not account for the survey design parameters as these were not consistently available across all surveys.

Among those with excess PHA, we run mixed effects logistic regression models to assess which socio-demographic (level of education, marital and work status) and health-related (abdominal obesity, high cholesterol, smoking, and diabetes) variables were linked to being eligible for antihypertensive or lipid-lowering therapy. This analysis only included those with a PHA higher than their chronological age.

### Ethics

This study used nationally representative survey data that are in the public domain and can be requested through the online repository (https://extranet.who.int/ncdsmicrodata/index.php/home).

### Role of the funding source

The funder had no role in the study design, analysis, interpretation, or decision to publish. The authors are collectively responsible for the accuracy of the data. The arguments and opinions in this work are those of the authors alone, and do not represent the position of the institutions to which they belong.

## Results

### Data description

The pooled dataset included 94,655 individuals from 41 countries in six world regions surveyed between 2013 and 2019. The weighted distribution of each component of the non-laboratory Framingham cardiovascular risk equation is described by country in the Table 1. Overall, the mean age ranged from 42 years (Tajikistan) to 48 years (Georgia, Belarus, and Morocco), whereas the proportion of men went from 29.8% (Sao Tome and Principe) to 65.8% (Timor-Leste). The mean SBP ranged from 120.5 mmHg (Jordan) to 139.7 mmHg (Tuvalu). The lowest mean BMI was 20.7 kg/m^2^ (Ethiopia) and the highest mean BMI was 35.6 kg/m^2^ (Nauru). The prevalence of diabetes went from 1.8% (Timor-Leste) to 55.6% (Tokelau) and the proportion of smokers ranged from 4.7% (Turkmenistan) to 56.5% (Tokelau).

**Table 1:** Weighted distribution of cardiovascular risk factors in each survey.

Region	Country	Year	Sample size	Age (years; mean and 95% CI)	Proportion of men (%)	SBP (mmHg; mean and 95% CI)	BMI (kg/m2; mean and 95% CI)	Proportion (%) of smokers (95% CI)	Proportion (%) of people with diabetes (95% CI)
**Africa**	Algeria	2017	4219	45 (44-45)	50.9	129.6 (128.8-130.5)	27.4 (27.2-27.6)	15 (13.8-16.4)	13.6 (12.3-14.9)
**Africa**	Benin	2015	3070	43 (41-44)	43.5	128.3 (124.7-131.9)	23.6 (23-24.2)	5.7 (4.2-7.7)	7.1 (5-10)
**Africa**	Botswana	2014	1900	44 (43-45)	49.9	131.4 (130.1-132.8)	24.8 (24.4-25.3)	22.3 (18.1-27.1)	6 (4.6-7.9)
**Africa**	Eswatini	2014	1402	45 (44-45)	44.5	129.5 (128.1-130.9)	27.6 (27-28.1)	8.4 (6.4-10.9)	9.5 (7.8-11.6)
**Africa**	Ethiopia	2015	4907	43 (42-43)	55.6	123.3 (122.4-124.2)	20.7 (20.5-20.9)	5.7 (4.7-6.9)	3.2 (2.6-4)
**Africa**	Kenya	2015	2466	43 (43-44)	50.5	128.4 (127.1-129.7)	23.9 (23.3-24.5)	11.8 (9.4-14.6)	4.3 (3.1-6)
**Africa**	Malawi	2017	2300	44 (43-44)	49	122.7 (121.2-124.2)	23 (22.7-23.3)	15.7 (12.1-20.1)	2.1 (1.4-3.3)
**Africa**	Sao Tome and Principe	2019	1155	43 (42-44)	29.8	130.5 (128.9-132)	25.9 (25.5-26.3)	4.9 (3.7-6.5)	14.5 (12.3-17)
**Africa**	Uganda	2014	1794	44 (43-45)	51.1	127.4 (126-128.7)	22.7 (22.4-23)	14.3 (11.8-17.2)	2.5 (1.7-3.7)
**Africa**	Zambia	2017	1958	43 (42-43)	48.9	126.3 (125.3-127.2)	23.8 (23.4-24.1)	14.5 (12.4-16.8)	10.8 (9.1-12.9)
**Americas**	Ecuador	2018	2632	47 (47-48)	48	122.1 (121-123.1)	28 (27.8-28.2)	12.8 (10.5-15.4)	13.9 (12.3-15.7)
**Americas**	Guyana	2016	543	46 (45-47)	45.4	129.4 (127.7-131.1)	28.1 (27.4-28.8)	12.2 (9.2-16.1)	23.2 (19.1-27.7)
**Eastern Mediterranean**	Afghanistan	2018	1924	44 (43-45)	55.6	129.5 (127.9-131)	26 (25.5-26.5)	9.2 (6.7-12.5)	15.4 (12.3-19.1)
**Eastern Mediterranean**	Iraq	2015	2430	45 (45-46)	48.5	133.4 (132.6-134.3)	30.2 (29.8-30.5)	20.7 (18.4-23.2)	18.6 (16.6-20.8)
**Eastern Mediterranean**	Jordan	2019	2111	45 (45-46)	41.2	120.5 (119.4-121.6)	30.2 (29.8-30.6)	35.8 (32.8-39)	17.3 (15.2-19.6)
**Eastern Mediterranean**	Kuwait	2014	1168	44 (43-45)	47.4	123.5 (122.6-124.4)	30.7 (30.4-31)	20.1 (17.7-22.8)	21.6 (19.2-24.1)
**Eastern Mediterranean**	Kyrgyzstan	2013	1789	44 (44-45)	51	134.2 (132.7-135.6)	27.1 (26.7-27.5)	25.8 (22.4-29.4)	7.5 (6.1-9.3)
**Eastern Mediterranean**	Lebanon	2017	903	46 (44-47)	39.5	129.1 (125.5-132.7)	28.3 (27.8-28.8)	34.5 (29.4-39.9)	13.6 (10.8-16.9)
**Eastern Mediterranean**	Morocco	2017	3404	48 (47-48)	49	131.6 (130.9-132.3)	27 (26.8-27.2)	12.2 (10.9-13.7)	16.6 (15.3-17.9)
**Eastern Mediterranean**	Sudan	2016	4396	44 (43-44)	53.2	131.3 (130.5-132.1)	24.2 (23.9-24.5)	9 (7.8-10.4)	10.9 (9.5-12.5)
**Europe**	Armenia	2016	1032	47 (46-48)	46.7	133.8 (132.1-135.5)	27.4 (26.9-27.9)	27.6 (23.7-31.8)	9.7 (7.2-12.9)
**Europe**	Azerbaijan	2017	1852	46 (45-46)	48.7	129.1 (127.9-130.4)	27.7 (27.3-28)	25.5 (23-28.2)	8.8 (7.4-10.4)
**Europe**	Belarus	2017	3690	48 (47-48)	46.6	137.1 (136.2-138.1)	27.8 (27.5-28)	29.9 (28-31.9)	7.5 (6.5-8.6)
**Europe**	Georgia	2016	1968	48 (48-49)	42.5	132.4 (131.2-133.7)	29.2 (28.8-29.5)	26 (23.4-28.7)	7.7 (6.4-9.2)
**Europe**	Republic of Moldova	2013	2082	46 (46-47)	52.2	136.9 (135.5-138.2)	27.6 (27.3-27.9)	26.1 (23.5-29)	10.2 (8.7-12)
**Europe**	Tajikistan	2017	1699	42 (41-43)	54	134 (132.3-135.6)	26.9 (26.6-27.3)	5.8 (4-8.2)	8.3 (6.6-10.3)
**Europe**	Turkmenistan	2018	2523	44 (44-45)	52.3	130.3 (129.3-131.3)	26.5 (26.2-26.7)	4.7 (3.6-6.1)	6.6 (5.5-8)
**Southeast Asia**	Bangladesh	2018	4580	46 (46-47)	51.7	123.1 (122-124.1)	22.7 (22.4-22.9)	28.3 (26.4-30.3)	12.5 (11-14.2)
**Southeast Asia**	Bhutan	2019	3547	44 (43-44)	56.8	127 (126-127.9)	25.7 (25.5-25.9)	7.5 (5.9-9.4)	5.9 (4.9-7.1)
**Southeast Asia**	Myanmar	2014	6216	44 (44-45)	51.4	127.2 (124.6-129.8)	22.8 (22.3-23.4)	27.3 (25.1-29.5)	7.4 (5.1-10.5)
**Southeast Asia**	Nepal	2019	3523	45 (45-46)	47.2	127.5 (126.5-128.5)	23.4 (23.1-23.6)	20.8 (18.5-23.3)	8.5 (6.7-10.7)
**Southeast Asia**	Sri Lanka	2015	3117	47 (46-47)	49.3	128.8 (127.9-129.6)	23.5 (23.3-23.7)	16.3 (14.7-17.9)	16.9 (15.4-18.6)
**Southeast Asia**	Timor-Leste	2014	1683	46 (46-47)	65.8	131.8 (127.7-135.9)	21.9 (21.4-22.4)	54.8 (39-69.6)	1.8 (0.6-5.3)
**Western Pacific**	Brunei Darussalam	2016	1218	45 (44-46)	48.3	128.8 (127.3-130.2)	28.3 (27.9-28.7)	18.5 (15.4-22)	18.4 (15.5-21.7)
**Western Pacific**	Kiribati	2016	697	46 (44-48)	43.7	130.3 (128.2-132.4)	30.9 (30.1-31.8)	54 (45.6-62.2)	23.8 (20.3-27.7)
**Western Pacific**	Mongolia	2019	3924	44 (44-45)	49.9	124.3 (123.6-125)	27 (26.8-27.2)	28.5 (26.7-30.3)	12.4 (11.2-13.8)
**Western Pacific**	Nauru	2016	446	43 (43-44)	51.8	126.6 (125.3-127.9)	35.6 (35.1-36.1)	42 (39-45.1)	30.1 (25.8-34.8)
**Western Pacific**	Solomon Islands	2015	1131	44 (43-45)	48.1	124.9 (123.1-126.8)	27.3 (26.9-27.8)	35 (29.9-40.5)	7.6 (5.9-9.8)
**Western Pacific**	Tokelau	2014	319	45 (44-47)	46.2	130.5 (128.8-132.2)	34.6 (34.2-35.1)	56.5 (43.8-68.4)	55.6 (47.4-63.5)
**Western Pacific**	Tuvalu	2015	636	47 (46-49)	51.6	139.7 (136.8-142.5)	33.8 (33.1-34.6)	35.1 (26.8-44.4)	18.8 (14.8-23.6)
**Western Pacific**	Vietnam	2015	2266	46 (45-46)	49.6	123.1 (121.9-124.2)	22.4 (22.2-22.6)	29.7 (27.2-32.3)	4.3 (3.5-5.4)

In a sensitivity analysis restricted to the population with measured HDL cholesterol, we observed no big differences between mean PHA computed as per the laboratory and non-laboratory risk scores in both men (53 vs 55; *p<*0.05) and women (50 vs 49; *p<*0.05) (Supplementary Table 2).

### Differences between chronological and predicted heart age

**Figure 1:**
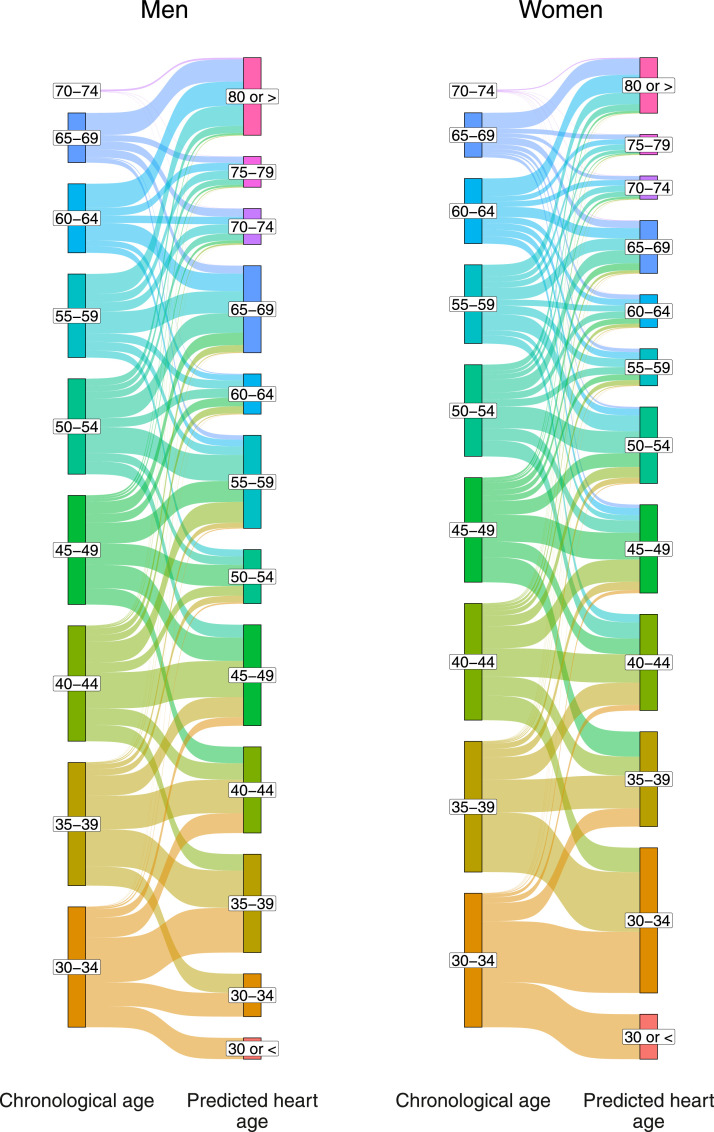
**Distribution of predicted heart age (PHA) groups by chronological age groups, by sex**. Similar figures by world region are shown as Supplementary Figures 2–7.

In the pooled dataset, there was high divergence between the chronological and the PHA (Figure 1). In the pooled dataset, 36% of those aged 30-34 years (45% in Americas vs 28% in Western Pacific) had a PHA of 30-34 years, whereas only 9% of those aged 60–64 years (11% in Africa vs 7% in Europe and Western Pacific) had a PHA of 60-64 years. Although people aged 40-44 years had PHAs as low as 30–34 years, they also had PHAs as high as ≥80 years. The proportion of people with PHA higher than their chronological age increased with older age groups; although this finding was consistent across regions, proportions varied by region (Supplementary Figures 3–8). In the pooled dataset, while 36% of people aged 30-34 years had higher PHA groups, 69% of those aged 60-64 years had higher PHA groups.

In those younger than 40 years old (30-39 years), the proportion of people with PHAs higher than 40 years was 37% in Western Pacific, 31% in Europe, 30% in Eastern Mediterranean, 25% in Southeast Asia, 22% in the Americas, and 21% in Africa.

### Distribution of predicted heart age categories

**Figure 2:**
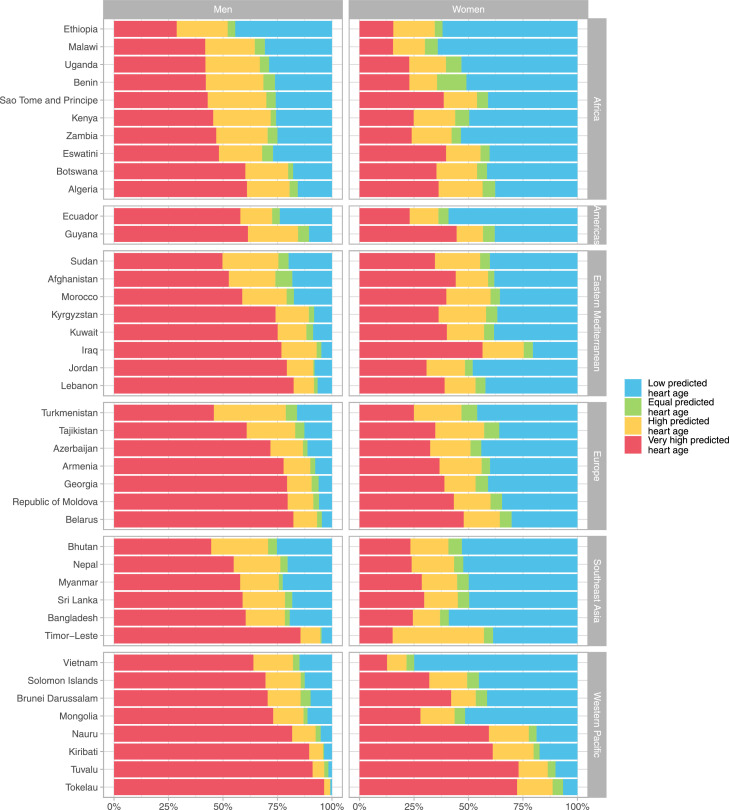
**Proportion (%) of low, equal, high, and very high predicted heart age (PHA) by country and sex, stratified by region**. Low predicted heart age (PHA) refers to a PHA lower than the chronological age (i.e., PHA – chronological age < 0). Equal PHA refers to a PHA equal to the chronological age (i.e., PHA – chronological age = 0). High predicted heart age (PHA) refers to an excess PHA between 1 to 4 years (i.e., PHA – chronological age ≥ 1 and ≤ 4). Very high predicted heart age (PHA) refers to an excess PHA exceeding 5 years (i.e., PHA – chronological age ≥ 5).

The prevalence of each PHA category by country and sex are shown in Figure 2. Across all countries, the prevalence of low PHA was higher in women than men. In men, the countries with the highest prevalence of low PHA were Ethiopia (44.4%), Malawi (30.8%), and Uganda (28.8%); all these countries are in Africa. In women, these countries were Vietnam (74.9%), Malawi (64.1%) and Ethiopia (61.9%); the latter two countries are in Africa. Conversely, in both sexes, the countries with the lowest prevalence of low PHA were Tokelau (0.7% in men, 6.6% in women), Tuvalu (1.6% in men, 10.1% in women), and Kiribati (3.6% in men, 17.4% in women); these three countries are in Western Pacific. The countries with the highest gender disparity in the prevalence of low PHA were Vietnam (14.9% in men vs 74.9% in women), Mongolia (11.2% in men vs 51.6% in women), and Jordan (8.0% in men vs 48.1% in women).

The prevalence of very high PHA was higher in men than women across all countries. In both sexes, the countries with the highest prevalence of very high PHA were Tokelau (96.4% in men, 72.3% in women), Tuvalu (91.1% in men, 73.0% in women), and Kiribati (89.5% in men, 61.1% in women); all these countries are in Western Pacific. On the other hand, in men, countries with the lowest prevalence of very high PHA were Ethiopia (28.7%), Malawi and Uganda (both with 41.9%), and Benin (42.1%); all these countries are in Africa. In women, countries with the lowest prevalence of very high PHA were Vietnam (12.7%), Timor-Leste (15.2%), and Malawi (15.4%); these countries did not belong to the same region. The countries with the highest gender disparity in the prevalence of very high PHA were Timor-Leste (85.5% in men vs 15.2% in women), Vietnam (64.0% in men vs 12.7% in women), and Jordan (79.3% in men vs 30.8% in women).

In 34 countries (out of 41), more than 50% of people with very high PHA were <50 years old. The countries with the lowest proportions of people with very high PHA that were <50 years old were Georgia (42%), Morocco and Armenia (both with 46%), and Belarus, Turkmenistan, Ecuador, and Sri Lanka (all with 47%); four of these seven countries were in Europe. Conversely, countries with the highest proportions of people with very high PHA that were <50 years old were Zambia (68%), Nauru (67%), and Malawi (66%); two of these countries are in Africa.

**Figure 3:**
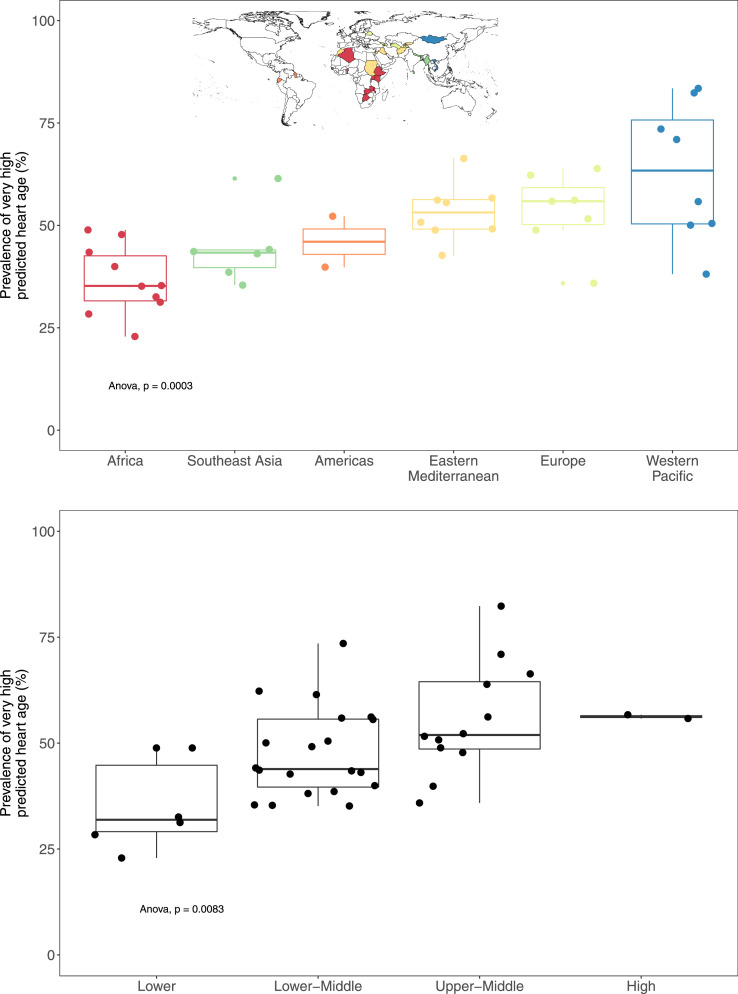
**Proportion (%) of very high predicted heart age (PHA) by world region and country income**. Each dot is a country. The centre line denotes the median prevalence (50th percentile) of very high predicted heart age (PHA) by region and income, while the boxes contain the 25th to 75th percentiles. The whiskers mark the 5th and 95th percentiles. Colours represent the world region in which countries are included. Very high PHA refers to an excess PHA exceeding 5 years (i.e., PHA – chronological age ≥ 5). p values for one-way ANOVA tests.

There were marked differences in the prevalence of very high PHA by world region (*p<*0.05 for one-way ANOVA test) and income (*p<*0.05 for one-way ANOVA test). When countries were grouped by world regions, the prevalence of very high PHA was highest in Western Pacific and lowest in Africa (Figure 3). We observed a positive correlation between the prevalence of very high PHA and income group; that is, the prevalence of very high PHA in low-income countries was lower than in high- and upper-middle-income countries. Aggregated by year (2012-2019), countries with more recent data had lower prevalence estimates of very high PHA compared with countries with older data (Supplementary Figure 9).

When population was stratified by their absolute CVD risk (Supplementary Figure 10), the prevalence of very high PHA increased with higher quintiles of CVD risk, whereas the prevalence of low PHA increased with lower quintiles of CVD risk. In the highest quintile of CVD risk, the prevalence of very high PHA was >80% in all countries except for Ethiopia (40 out of 41), whereas in the lowest quintile of CVD risk, the prevalence of very high PHA was <12% in all countries except for Tokelau (40 out of 41 countries).

### Eligibility for treatment in people with excess predicted heart age

**Figure 4:**
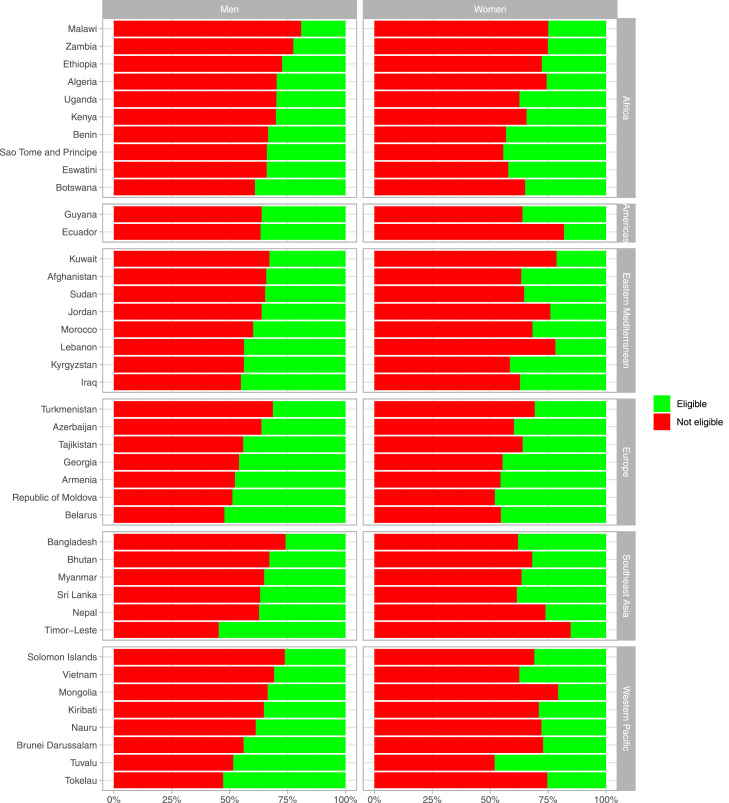
**Proportion (%) of people with excess predicted heart age (PHA) who are eligible/not eligible for antihypertensive treatment based on guideline recommendations**. Excess predicted heart age (PHA) refers to a positive difference between predicted heart age and chronological age (i.e., PHA – chronological age > 0). Similar figures for people with an excess PHA exceeding 5, 10, and 20 years are shown as Supplementary Figures 9–11. Countries in the same region are presented in ascending order based on their proportion of eligible men.

In 37 (out of 41) countries, less than 50% of men with excess PHA were eligible for antihypertensive treatment (Figure 4). Countries with the lowest proportions of men with excess PHA that were eligible for antihypertensive treatment were Malawi (19.0%), Zambia (22.5%), and Bangladesh (25.9%); the first two countries are in Africa. In all countries, less than 50% of women with excess PHA were eligible for antihypertensive treatment. The lowest proportions in women were in Timor-Leste (15.2%), Ecuador (18.1%), and Mongolia (20.7%). On the other hand, among those without excess PHA, <5% of people were eligible for antihypertensive therapy in all countries in men and nearly all (40 out of 41) countries in women.

**Figure 5:**
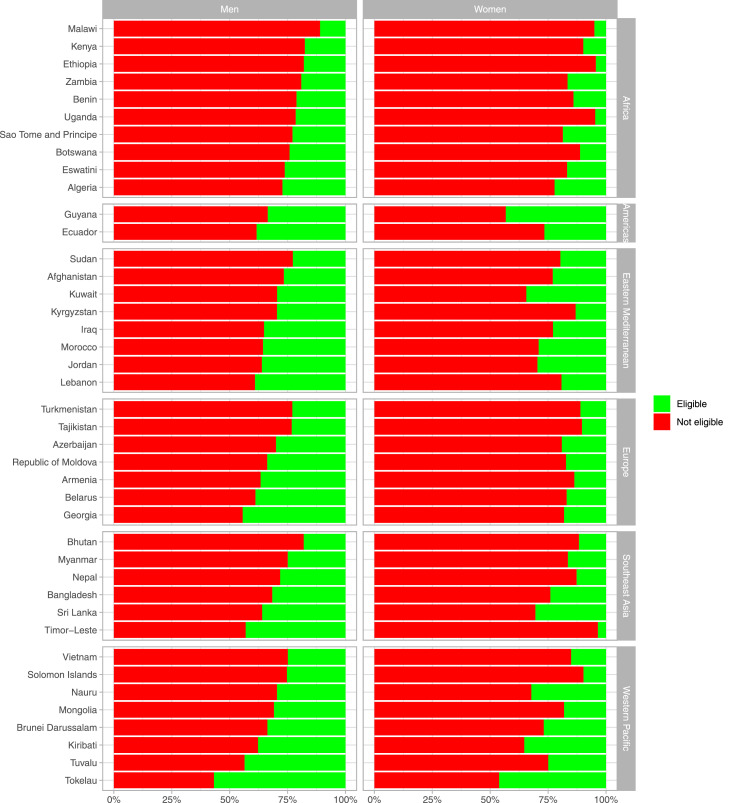
**Proportion (%) of people with excess predicted heart age (PHA) who are eligible/not eligible for lipid-lowering treatment based on guideline recommendations**. Excess predicted heart age (PHA) refers to a positive difference between predicted heart age and chronological age (i.e., PHA – chronological age > 0). Similar figures for people with an excess PHA exceeding 5, 10, and 20 years are shown as Supplementary Figures 12–14. Countries in the same region are presented in ascending order based on their proportion of eligible men.

In 40 (out of 41) countries, less than half of men with excess PHA were eligible for lipid-lowering treatment (Figure 5). The lowest proportions of men with excess PHA that were eligible for lipid-lowering treatment were in Malawi (11.0%), Kenya (17.6%), and Ethiopia and Bhutan (both with 18.0%); the first three countries are in Africa. In all countries, less than 50% of women with excess PHA were eligible for lipid-lowering treatment. The lowest proportions in women were in Timor-Leste (3.5%), Ethiopia (4.4%), and Uganda (4.7%). Of note, among those without excess PHA, <3% of people were eligible for lipid-lowering therapy in all countries in both men and women.

The proportion of people with excess PHA that were eligible for antihypertensive or lipid-lowering treatment improved with higher excesses of PHA (e.g., ≥10 years) (Supplementary Figures 11–16). In all countries for antihypertensive treatment and in 39 (out of 41) countries for lipid-lowering treatment, ≥70% of men with a PHA exceeding their chronological age by ≥20 years were eligible for drug treatment (Supplementary Figures 13 and 16). In 35 (out of 41) countries for antihypertensive treatment and in 7 (out of 41) countries for lipid-lowering treatment, ≥70% of women with a PHA exceeding their chronological age by ≥20 years were eligible for drug treatment.

### Potential correlates of excess predicted heart age and eligibility for therapy

Having tertiary education and paid employment were both associated with a lower probability of having PHA excess (Table 2). High waist circumference, high total cholesterol, and specially smoking and having (either-self-reported or biomarker-based) diabetes were all associated with higher odds of having PHA excess.

**Table 2:** Multilevel regression models for excessive predicted heart age.

Variable	OR (95% CI)	*p* value
**Education (Referent: None)**		
**Some primary/primary**	1.1 (1.05-1.15)	<0.0001
**Secondary/high**	1.1 (1.05-1.16)	0.0002
**University**	0.96 (0.9-1.03)	0.2638
**Abdominal obesity (Referent: Normal waist circumference)**	2.19 (2.11-2.27)	<0.0001
**High cholesterol (Referent: Normal)**	1.38 (1.33-1.44)	<0.0001
**Marital status (Referent: Single)**		
**Married/cohabiting**	1.23 (1.15-1.31)	<0.0001
**Divorced/Separated/Widowed**	0.97 (0.9-1.04)	0.4205
**Work status (Referent: Unemployed)**		
**Employed and paid**	0.94 (0.9-0.99)	0.0197
**Employed but not paid**	0.88 (0.78-0.99)	0.0344
**Homemaker**	0.52 (0.49-0.55)	<0.0001
**Student**	0.67 (0.5-0.91)	0.0113
**Smoker (Referent: Non-smoker)**	18.89 (17.5-20.39)	<0.0001
**With diabetes, (Referent: Not with diabetes)**	28.25 (24.93-32.02)	<0.0001
**Random effects, country: region (variance)**	0.14	
**Random effects, region (variance)**	0.01	

*Regression models were adjusted for socio-demographic (level of education, marital, and work status) and health-relates (abdominal obesity, high cholesterol, smoking, and diabetes) variables.

Among those with excess PHA, formal education (compared to no education) and being employed (compared to being unemployed) were both associated with lower odds of needing antihypertensive and lipid-lowering therapy (Table 3). In contrast, having abdominal obesity and high total cholesterol were both associated with higher odds of being eligible of antihypertensive and lipid-lowering therapy. Having diabetes (either-self-reported or biomarker-based) was associated with higher odds of being eligible of antihypertensive therapy, whereas smoking increased the odds of being eligible of lipid-lowering therapy.

**Table 3:** Multilevel regression models for eligibility for antihypertensive and lipid-lowering therapy among those with excessive predicted heart age.

	Eligibility for antihypertensive therapy		Eligibility for lipid-lowering therapy	
Variable	OR (95% CI)	*p* value	OR (95% CI)	*p* value
**Education (Referent: None)**				
**Some primary/primary**	0.74 (0.7-0.78)	<0.0001	0.85 (0.8-0.91)	<0.0001
**Secondary/high**	0.62 (0.59-0.66)	<0.0001	0.79 (0.74-0.85)	<0.0001
**University**	0.6 (0.55-0.64)	<0.0001	0.83 (0.76-0.9)	<0.0001
**Abdominal obesity (Referent: Normal waist circumference)**	1.43 (1.37-1.49)	<0.0001	1.35 (1.29-1.41)	<0.0001
**High cholesterol (Referent: Normal)**	1.41 (1.35-1.47)	<0.0001	1.38 (1.32-1.45)	<0.0001
**Marital status (Referent: Single)**				
**Married/cohabiting**	1.54 (1.43-1.67)	<0.0001	2.28 (2.07-2.51)	<0.0001
**Divorced/Separated/Widowed**	2.21 (2.02-2.42)	<0.0001	2.71 (2.44-3.01)	<0.0001
**Work status (Referent: Unemployed)**				
**Employed and paid**	0.47 (0.45-0.5)	<0.0001	0.41 (0.39-0.44)	<0.0001
**Employed but not paid**	0.58 (0.51-0.67)	<0.0001	0.55 (0.48-0.64)	<0.0001
**Homemaker**	0.4 (0.37-0.42)	<0.0001	0.35 (0.33-0.37)	<0.0001
**Student**	0.44 (0.29-0.65)	<0.0001	0.46 (0.3-0.71)	0.0005
**Smoker (Referent: Non-smoker)**	1.01 (0.97-1.06)	0.5839	1.71 (1.63-1.8)	<0.0001
**With diabetes, (Referent: Not with diabetes)**	1.84 (1.75-1.93)	<0.0001	-	-
**Random effects, country: region (variance)**	0.09		0.08	
**Random effects, region (variance)**	0.03		0.08	

*Regression models were adjusted for socio-demographic (level of education, marital, and work status) and health-relates (abdominal obesity, high cholesterol, smoking, and diabetes) variables. Diabetes was not included in the regression model for eligibility for lipid-lowering therapy because all adults ≥40 years with diabetes are eligible for lipid-lowering therapy following the WHO guidelines.

## Discussion

In this work with 94,655 adults without a history of CVD, we provided population-based estimates of PHA in 41 countries. We observed that, regardless of the world region, the PHA was substantially higher than chronological age by as much as 40 years. In general, the proportion of people with excess PHA increased with older chronological age. There was high inter-country variability in the prevalence of low PHA and very high PHA. Across all countries, men had higher prevalence of very high PHA and lower prevalence of low PHA compared to women. Countries with the highest prevalence low PHA and the lowest prevalence of very high PHA were in Africa; for example, 3 out of 10 men and 2 out of 10 women had very high PHA in Ethiopia. Conversely, countries in the Western Pacific had the lowest prevalence of low PHA and the highest prevalence of very high PHA; alarmingly, 9 out of 10 men and 7 out of 10 women had very high PHA in Tokelau and Tuvalu. In 34 (out of 41) countries, most people with very high PHA were <50 years old. In nearly all countries for men and in all countries for women, more than half of the population with excess PHA were not eligible for antihypertensive nor lipid-lowering treatment based on the WHO guidelines.^10^ Male sex, having older chronological age, abdominal obesity, and high total cholesterol were all associated with higher odds of having excess PHA, whereas having tertiary education and being employed were inversely associated with excess PHA.

Emerging evidence indicates that PHA could have potential benefits in clinical practice.^15^ For example, the concept of (high) absolute cardiovascular risk is better understood by patients when expressed as PHA.^15^ Furthermore, communicating patients’ absolute cardiovascular risk as PHA elicits more emotional impact and risk perception than absolute risk predictions.^15^^,^^29^ Compared to usual care, complex interventions that include PHA (e.g., plus counselling) result on larger reductions of risk factors levels and CVD risk.^15^^,^^30^ The latter finding is important in terms of public health, as the prevalence of CVD risk factors is still increasing in LMICs,^2^^,^^3^^,^^31^ and new approaches to communicate CVD risk could help to reduce this upward trend. Despite these potential benefits, the use of PHA on clinical recommendations is scarce and limited to high-income countries.^20^^,^^32^ In this line, our results could spark interest from global or regional organizations, especially those in LMICs, to assess whether PHA could be included on clinical practice guidelines of cardiovascular risk assessment (e.g., HEARTS^10^). Furthermore, there are no large-scale interventions that target CVD reduction expressing CVD risk as predicted heart age (PHA). Current evidence suggests that expressing CVD risk as PHA could have benefits in reducing CVD risk along with individual CVD risk factors.^15^ Informing the PHA, alone or together with the absolute cardiovascular risk, needs to be further tested in population-wide interventions including middle-aged adults, amongst whom the PHA is generally higher than their chronological age.

Population-based estimates of PHA are useful to inform which populations (e.g., countries in the Western Pacific) would benefit the most out of this novel approach. To date, most population-based estimates come from high-income countries^17^^,^^18^^,^^23^ or few LMICs.^19^ Even though our findings and prior work indicate that PHA increases with older chronological age,^23^ we found that people with very high PHA were mostly younger than 50 years old. In some countries, even 7 out of 10 people with very high PHA were <50 years old. This finding is of special interest, as young adults with CVD risk factors could have low predictions of absolute CVD risk (because of their chronological age),^16^ which could make them underestimate their true risk and not adopting healthy lifestyle choices. Furthermore, we observed regional differences in PHA among young adults. In those younger than 40 years old, almost 4 out of 10 people in Western Pacific had PHAs higher than 40 years, whereas only 2 out of 10 people in Africa and the Americas had PHAs higher than 40 years.

Even though PHA has been included in guidelines for the prevention of CVDs,^20^ there is great concern about its use to guide preventive treatment.^16^ To date, most guidelines for the prevention of CVDs recommend to begin preventive treatment based on specific thresholds of absolute cardiovascular risk (i.e., risk-based approach).^10^, ^11^, ^12^, ^13^^,^^33^ Nonetheless, the third iteration of the Joint British Societies (JBS-3) guidelines recommend that medication should be considered in people with any excess of PHA,^20^ and these people could have low absolute CVD risk. The latter could result in mass medicalization of low-risk people that would not benefit of treatment. Our results suggest that most people with excess PHA are not eligible for antihypertensive nor lipid-lowering treatment following the risk-based approach recommended by the WHO. In this line, socio-economic variables could help predicting which people could have excess PHA, and which people with excess PHA could be eligible/not eligible for therapy. These findings must be confirmed with PHA calculators developed by the WHO along with prospective studies analysing the benefit of preventive treatment (with or without antiplatelet drugs^34^) in people with excess PHA (regardless of having low absolute CVD risk). Overtreating low-risk people could end in higher costs for both the health system and patients, which is of special importance in LMICs where the availability and affordability of drugs is lower than HICs.^35^^,^^36^ It should also be noted that, regardless of their absolute CVD risk (and therefore PHA), all people should receive lifestyle counselling (i.e., healthy diet, physical activity, smoking cessation, and avoiding the harmful use of alcohol).^10^

The study most similar to ours (i.e., same CVD risk equation and focused on LMICs) produced national estimates of PHA in 7 countries (6 LMICs) following the non-laboratory Framingham risk score. Similar to our findings, Appiah and Capistrant reported that PHA was substantially higher than chronological age across all countries.^19^ In their regression models, they also reported that tertiary education was associated with lower odds of having excess PHA, whereas abdominal obesity was associated with higher odds of having excess PHA.^19^ Although their study produced the first population-based evidence of PHA across few LMICs, we advanced this evidence by: 1) Including a more diverse population as we studied 41 countries (38 LMICs) in 6 world regions; and 2) Describing the eligibility of antihypertensive and lipid-lowering treatment in those with excess PHA. Furthermore, we run regression models to assess correlates for being eligible for drug treatment in those with excess PHA. In so doing, we found that marital status was the strongest predictor for being eligible for antihypertensive and lipid-lowering therapy: compared to single people, being married/cohabiting and being divorced/separated/widowed doubled the odds of being eligible for therapy. This could be explained by the fact that single people were younger (e.g., mean age: 42 years in single people vs 55 years in widowed people) and had lower levels of CVD risk factors (e.g., mean SBP: 127 mmHg in single people vs 136 mmHg in widowed people).

Prior population-based studies mainly come from high-income countries. A web-based study in users of a heart age tool from 13 countries (only 2 LMICs) found that, in general, PHA exceeded chronological age.^24^ They also reported that women had lower PHA than men. Both findings are consistent with our results. Of note, while their study used self-reported data on anthropometric measures and biomarkers,^24^ ours used objectively measured risk factors. A national study in the United States (US) reported that 5 out of 10 men and 4 out of 10 women had very high PHA^23^; compared to these, our estimates of very high PHA were higher in 30 (out of 41) countries in men and 11 (out 41) countries in women.

Leveraging on nationally-representative surveys that followed a standard protocol,^21^ we produced population-based estimates of PHA in multiple countries, and advanced previous literature by describing the proportion of the population with excess PHA that would need antihypertensive and lipid-lowering treatment. Furthermore, in contrast with previous research,^19^^,^^23^ CVD risk factors included in the PHA calculation were directly measured (i.e., not modelled nor self-reported only) and accounted for total diabetes (i.e., both diagnosed and undiagnosed). Nonetheless, there are limitations we must acknowledge. First, we used the non-laboratory model of the Framingham risk score to produce our PHA estimates. As the Framingham non-laboratory risk score overestimates the CVD risk compared with the Framingham laboratory risk score,^37^ our estimates of PHA could have been overestimated. Nonetheless, we observed no big differences between PHA computed using the laboratory vs non-laboratory risk scores in the sensitivity analysis. Second, because not all surveys measured other specific biomarkers (e.g., HDL), we could not compute PHA as per other risk scores (e.g., pooled cohort equations^38^); the latter was also limited by the lack of algorithms to produce PHA in novel CVD risk scores (e.g., 2019 WHO CVD risk charts^6^). Despite both limitations, it should be noted that most of the published literature regarding the effects of interventions using PHA have used the Framingham risk score to calculate PHA.^15^ Machine learning techniques could also provide PHA estimates across multiple countries, as they could leverage on simple predictors that are routinely available in national surveys. Third, as we used cross-sectional data to produce PHA estimates, long-term outcomes that could be related with excess PHA (e.g., mortality) could not be studied. Future studies with prospective data are needed to test to what degree PHA is related to CVD events or CVD mortality. Fourth, although we used objectively measured variables to compute PHA in people without history of CVD, CVD history itself was assessed using a self-reported question. Research has shown that people tend to over-report CVDs and thus, assessing medical records would have been ideal to reduce self-reporting bias on the history of CVDs.^39^ Finally, although we leveraged on nationally representative survey data, applying our selection criteria reduced the sample size included in the analysis from some countries. Arguably, the missing data could be considered missing at random with some participants refusing to take some tests, without this decision being associated with the risk factor or outcome; similarly, potential laboratory errors leading to implausible values (which were dropped) may have been at random as well. Nonetheless, our results should be interpreted considering the reduction in sample size. Where the reduction was substantial, the results should be cautiously interpreted as nationally representative; in such cases, it may be reasonable to regard the results as information of the general population warranting further verification with a larger sample size.

Although large disparities in the distribution of PHA exist between world regions, PHA is generally higher than chronological age, specially across middle-aged adults. Whether PHA should be used to guide preventive treatment for CVDs remains under debate, and our results suggest that most people with excess PHA would not be eligible for antihypertensive nor lipid-lowering medication, but this could increase with higher excesses of PHA.

## Contributors

RMC-L conceived the idea with WCG-V. WCG-V conducted the analysis with support from GAQ-V. WCG-V wrote the first draft of the manuscript with support from GAQ-V and RMC-L. All authors provided a relevant scientific contribution and approved the submitted version.

## Data sharing statement

All data files are available from https://extranet.who.int/ncdsmicrodata/index.php/home. This data repository needs a username, password, and application. All data files are open access and released within days from the application.

## Declaration of interests

The authors declare no conflict of interest.

## Appendix Supplementary materials

Image, application 1

Image, application 2

Image, application 3

Image, application 4
