# Supplementary material for: Predicted heart age profile across 41 countries: A cross-sectional study of nationally representative surveys in six world regions
Source: eClinicalMedicine. 2022 Oct 1;52:101688. doi: 10.1016/j.eclinm.2022.101688 (PMC9596311; doi:10.1016/j.eclinm.2022.101688)
Supplement: Supplementary file 1 [file mmc1.docx]

**SUPPLEMENTARY MATERIAL**

**Predicted heart age profile across 41 countries: A cross-sectional study of nationally representative surveys in six world regions**

**Corresponding Author**

Rodrigo M Carrillo-Larco, MD

Department of Epidemiology and Biostatistics, School of Public Health, Imperial College London, London, UK.

[r.carrillo-larco@imperial.ac.uk](mailto:r.carrillo-larco@imperial.ac.uk)

[Supplementary Checklist. STROBE Checklist. 4](#_Toc113473827)

[Supplementary Figure 1. Flowchart of data cleaning. 7](#_Toc113473828)

[Supplementary Table 1. Flowchart of inclusion criteria by country. 8](#_Toc113473829)

[Supplementary Table 2. Mean predicted heart age as per the laboratory-risk score and non-laboratory-risk score. 10](#_Toc113473830)

[Supplementary Table 3. Protocol for blood pressure, glucose, and total cholesterol measurements by country. 10](#_Toc113473831)

[Supplementary Figure 2. Formula to estimate cardiovascular disease (CVD) risk using the Framingham risk score. 16](#_Toc113473832)

[Supplementary Figure 3. Distribution of predicted heart age (PHA) groups by chronological age groups in Africa, by sex 17](#_Toc113473834)

[Supplementary Figure 4. Distribution of predicted heart age (PHA) groups by chronological age groups in Americas, by sex 18](#_Toc113473835)

[Supplementary Figure 5. Distribution of predicted heart age (PHA) groups by chronological age groups in South-East Asia, by sex 19](#_Toc113473836)

[Supplementary Figure 6. Distribution of predicted heart age (PHA) groups by chronological age groups in Europe, by sex. 20](#_Toc113473837)

[Supplementary Figure 7. Distribution of predicted heart age (PHA) groups by chronological age groups in Eastern Mediterranean, by sex. 21](#_Toc113473838)

[Supplementary Figure 8. Distribution of predicted heart age (PHA) groups by chronological age groups in Western Pacific, by sex. 22](#_Toc113473839)

[Supplementary Figure 9. Proportion (%) of very high predicted heart age (PHA) by survey year. 23](#_Toc113473840)

[Supplementary Figure 10. Proportion (%) of low, equal, high, and very high predicted heart age (PHA) by country and quintile of absolute cardiovascular disease risk, stratified by region. 24](#_Toc113473841)

[Supplementary Figure 11. Proportion (%) of people with very high predicted heart age (PHA) who are eligible/not eligible for antihypertensive treatment based on guideline recommendations. 25](#_Toc113473842)

[Supplementary Figure 12. Proportion (%) of people with predicted heart age (PHA) exceeding 10 years who are eligible/not eligible for antihypertensive treatment based on guideline recommendations. 26](#_Toc113473843)

[Supplementary Figure 13. Proportion (%) of people with predicted heart age (PHA) exceeding 20 years who are eligible/not eligible for antihypertensive treatment based on guideline recommendations. 27](#_Toc113473844)

[Supplementary Figure 14. Proportion (%) of people with very high predicted heart age (PHA) who are eligible/not eligible for lipid-lowering treatment based on guideline recommendations. 28](#_Toc113473845)

[Supplementary Figure 15. Proportion (%) of people with predicted heart age (PHA) exceeding 10 years who are eligible/not eligible for lipid-lowering treatment based on guideline recommendations. 29](#_Toc113473846)

[Supplementary Figure 16. Proportion (%) of people with predicted heart age (PHA) exceeding 20 years who are eligible/not eligible for lipid-lowering treatment based on guideline recommendations. 29](#_Toc113473847)

# **Supplementary Checklist. STROBE Checklist.**

STROBE Statement—Checklist of items that should be included in reports of ***cross-sectional studies***

|  | Item No | Recommendation | Page No |
| --- | --- | --- | --- |
| **Title and abstract** | 1 | (*a*) Indicate the study’s design with a commonly used term in the title or the abstract | 1 |
|  |  | (*b*) Provide in the abstract an informative and balanced summary of what was done and what was found | 2 |
| Introduction | | | |
| Background/rationale | 2 | Explain the scientific background and rationale for the investigation being reported | 3-4 |
| Objectives | 3 | State specific objectives, including any prespecified hypotheses | 3-4 |
| Methods | | | |
| Study design | 4 | Present key elements of study design early in the paper | 4 |
| Setting | 5 | Describe the setting, locations, and relevant dates, including periods of recruitment, exposure, follow-up, and data collection | 4 |
| Participants | 6 | (*a*) Give the eligibility criteria, and the sources and methods of selection of participants | 4 |
| Variables | 7 | Clearly define all outcomes, exposures, predictors, potential confounders, and effect modifiers. Give diagnostic criteria, if applicable | 5-9 |
| Data sources/ measurement | 8* | For each variable of interest, give sources of data and details of methods of assessment (measurement). Describe comparability of assessment methods if there is more than one group | 5-7 |
| Bias | 9 | Describe any efforts to address potential sources of bias | 9-10 |
| Study size | 10 | Explain how the study size was arrived at | 4 |
| Quantitative variables | 11 | Explain how quantitative variables were handled in the analyses. If applicable, describe which groupings were chosen and why | 5-8 |
| Statistical methods | 12 | (*a*) Describe all statistical methods, including those used to control for confounding | 9 |
|  |  | (*b*) Describe any methods used to examine subgroups and interactions | 8-9 |
|  |  | (*c*) Explain how missing data were addressed | 4 |
|  |  | (*d*) If applicable, describe analytical methods taking account of sampling strategy | 8 |
|  |  | (*e*) Describe any sensitivity analyses | - |
| Results | | | |
| Participants | 13* | (a) Report numbers of individuals at each stage of study—eg numbers potentially eligible, examined for eligibility, confirmed eligible, included in the study, completing follow-up, and analysed | 10 |
|  |  | (b) Give reasons for non-participation at each stage | 4 |
|  |  | (c) Consider use of a flow diagram | 4 |
| Descriptive data | 14* | (a) Give characteristics of study participants (eg demographic, clinical, social) and information on exposures and potential confounders | 10 |
|  |  | (b) Indicate number of participants with missing data for each variable of interest | 4 |
| Outcome data | 15* | Report numbers of outcome events or summary measures | 10-15 |
| Main results | 16 | (*a*) Give unadjusted estimates and, if applicable, confounder-adjusted estimates and their precision (eg, 95% confidence interval). Make clear which confounders were adjusted for and why they were included | 10-15 |
|  |  | (*b*) Report category boundaries when continuous variables were categorized | 10-15 |
|  |  | (*c*) If relevant, consider translating estimates of relative risk into absolute risk for a meaningful time period | - |
| Other analyses | 17 | Report other analyses done—eg analyses of subgroups and interactions, and sensitivity analyses | 14 |
| Discussion | | | |
| Key results | 18 | Summarise key results with reference to study objectives | 15-16 |
| Limitations | 19 | Discuss limitations of the study, taking into account sources of potential bias or imprecision. Discuss both direction and magnitude of any potential bias | 18 |
| Interpretation | 20 | Give a cautious overall interpretation of results considering objectives, limitations, multiplicity of analyses, results from similar studies, and other relevant evidence | 16-17 |
| Generalisability | 21 | Discuss the generalisability (external validity) of the study results | 17-18 |
| Other information | | | |
| Funding | 22 | Give the source of funding and the role of the funders for the present study and, if applicable, for the original study on which the present article is based | 9,19 |

*Give information separately for exposed and unexposed groups.

**Note:** An Explanation and Elaboration article discusses each checklist item and gives methodological background and published examples of transparent reporting. The STROBE checklist is best used in conjunction with this article (freely available on the Web sites of PLoS Medicine at http://www.plosmedicine.org/, Annals of Internal Medicine at http://www.annals.org/, and Epidemiology at http://www.epidem.com/). Information on the STROBE Initiative is available at www.strobe-statement.org.

# **Supplementary Figure 1. Flowchart of data cleaning.**

30-74 years

278,586 people

75 countries

208,616 people

75 countries

Missing data on blood pressure, hypertension treatment, body mass index (BMI), diabetes, and smoking

188,591 people

74 countries

Implausible values in blood pressure, height, weight, BMI, waist circumference, fasting plasma glucose and total cholesterol

136,972 people

64 countries

136,944 people

64 countries

Non-pregnant women

94,655 people

41 countries

Without history of cardiovascular disease

**Supplementary Table 1. Flowchart of inclusion criteria by country.**

| **Country** | **Year** | **Initial sample size of people aged 30-74 years** | **Sample size excluding people with missing variables (% of initial sample size)** | **Sample size excluding people with missing variables and implausible values (% of initial sample size)** | **Sample size excluding people with missing variables, implausible values, and pregnant women (% of initial sample size)** | **Sample size excluding people with missing variables, implausible values, pregnant women, and those with history of CVD (% of initial sample size)** | **Final sample size** |
| --- | --- | --- | --- | --- | --- | --- | --- |
| Afghanistan | 2018 | 2426 | 2358 (97.2) | 2129 (87.8) | 2129 (87.8) | 1927 (79.4) | 1927 |
| Algeria | 2017 | 5481 | 5138 (93.7) | 4517 (82.4) | 4517 (82.4) | 4219 (77) | 4219 |
| Armenia | 2016 | 1832 | 1504 (82.1) | 1190 (65) | 1190 (65) | 1032 (56.3) | 1032 |
| Azerbaijan | 2017 | 2321 | 2157 (92.9) | 2038 (87.8) | 2038 (87.8) | 1852 (79.8) | 1852 |
| Bangladesh | 2018 | 6243 | 5923 (94.9) | 5228 (83.7) | 5228 (83.7) | 4580 (73.4) | 4580 |
| Belarus | 2017 | 4322 | 4302 (99.5) | 4058 (93.9) | 4058 (93.9) | 3691 (85.4) | 3691 |
| Benin | 2015 | 3492 | 3352 (96) | 3275 (93.8) | 3275 (93.8) | 3070 (87.9) | 3070 |
| Bhutan | 2019 | 4209 | 3784 (89.9) | 3662 (87) | 3660 (87) | 3547 (84.3) | 3547 |
| Botswana | 2014 | 2505 | 2417 (96.5) | 2035 (81.2) | 2035 (81.2) | 1900 (75.8) | 1900 |
| Brunei Darussalam | 2016 | 2824 | 1359 (48.1) | 1320 (46.7) | 1320 (46.7) | 1218 (43.1) | 1218 |
| Ecuador | 2018 | 3433 | 3329 (97) | 2923 (85.1) | 2923 (85.1) | 2632 (76.7) | 2632 |
| Eswatini | 2014 | 2091 | 1818 (86.9) | 1475 (70.5) | 1475 (70.5) | 1402 (67) | 1402 |
| Ethiopia | 2015 | 5841 | 5614 (96.1) | 5111 (87.5) | 5111 (87.5) | 4907 (84) | 4907 |
| Georgia | 2016 | 3595 | 3396 (94.5) | 2666 (74.2) | 2666 (74.2) | 1968 (54.7) | 1968 |
| Guyana | 2016 | 1932 | 1911 (98.9) | 606 (31.4) | 606 (31.4) | 543 (28.1) | 543 |
| Iraq | 2015 | 2919 | 2838 (97.2) | 2644 (90.6) | 2629 (90.1) | 2430 (83.2) | 2430 |
| Jordan | 2019 | 4111 | 3725 (90.6) | 2369 (57.6) | 2369 (57.6) | 2111 (51.4) | 2111 |
| Kenya | 2015 | 3002 | 2885 (96.1) | 2650 (88.3) | 2650 (88.3) | 2466 (82.1) | 2466 |
| Kiribati | 2016 | 1474 | 857 (58.1) | 767 (52) | 767 (52) | 697 (47.3) | 697 |
| Kuwait | 2014 | 2612 | 1932 (74) | 1260 (48.2) | 1260 (48.2) | 1168 (44.7) | 1168 |
| Kyrgyzstan | 2013 | 2233 | 2187 (97.9) | 2120 (94.9) | 2120 (94.9) | 1789 (80.1) | 1789 |
| Lebanon | 2017 | 1641 | 1490 (90.8) | 958 (58.4) | 958 (58.4) | 903 (55) | 903 |
| Malawi | 2017 | 2816 | 2751 (97.7) | 2524 (89.6) | 2524 (89.6) | 2300 (81.7) | 2300 |
| Mongolia | 2019 | 5181 | 5063 (97.7) | 4755 (91.8) | 4755 (91.8) | 3924 (75.7) | 3924 |
| Morocco | 2017 | 4122 | 4028 (97.7) | 3533 (85.7) | 3533 (85.7) | 3404 (82.6) | 3404 |
| Myanmar | 2014 | 7475 | 7165 (95.9) | 6934 (92.8) | 6933 (92.7) | 6216 (83.2) | 6216 |
| Nauru | 2016 | 850 | 664 (78.1) | 581 (68.4) | 581 (68.4) | 446 (52.5) | 446 |
| Nepal | 2019 | 4127 | 3896 (94.4) | 3592 (87) | 3592 (87) | 3523 (85.4) | 3523 |
| Republic of Moldova | 2013 | 3959 | 3375 (85.2) | 2621 (66.2) | 2620 (66.2) | 2082 (52.6) | 2082 |
| Sao Tome and Principe | 2019 | 1639 | 1562 (95.3) | 1209 (73.8) | 1208 (73.7) | 1155 (70.5) | 1155 |
| Solomon Islands | 2015 | 1876 | 1449 (77.2) | 1281 (68.3) | 1281 (68.3) | 1162 (61.9) | 1162 |
| Sri Lanka | 2015 | 4385 | 3881 (88.5) | 3322 (75.8) | 3322 (75.8) | 3117 (71.1) | 3117 |
| Sudan | 2016 | 5312 | 5057 (95.2) | 4487 (84.5) | 4487 (84.5) | 4396 (82.8) | 4396 |
| Tajikistan | 2017 | 1973 | 1946 (98.6) | 1825 (92.5) | 1825 (92.5) | 1699 (86.1) | 1699 |
| Timor-Leste | 2014 | 1936 | 1857 (95.9) | 1710 (88.3) | 1710 (88.3) | 1683 (86.9) | 1683 |
| Tokelau | 2014 | 374 | 361 (96.5) | 350 (93.6) | 349 (93.3) | 319 (85.3) | 319 |
| Turkmenistan | 2018 | 3037 | 2996 (98.6) | 2809 (92.5) | 2809 (92.5) | 2523 (83.1) | 2523 |
| Tuvalu | 2015 | 848 | 766 (90.3) | 719 (84.8) | 719 (84.8) | 636 (75) | 636 |
| Uganda | 2014 | 2371 | 2250 (94.9) | 1999 (84.3) | 1999 (84.3) | 1794 (75.7) | 1794 |
| Vietnam | 2015 | 3067 | 2558 (83.4) | 2507 (81.7) | 2507 (81.7) | 2266 (73.9) | 2266 |
| Zambia | 2017 | 2682 | 2510 (93.6) | 2044 (76.2) | 2044 (76.2) | 1958 (73) | 1958 |

**Supplementary Table 2. Mean predicted heart age as per the laboratory-risk score and non-laboratory-risk score.**

|  | **Sample size** | **Mean chronological age** | **Mean predicted heart age using the laboratory-risk-score** | **Mean predicted heart age using the non-laboratory-risk-score** | **p value for paired t-test between PHA using the laboratory-risk-score and PHA using the non-laboratory-risk-score** | **p value for paired t-test between chronological age and PHA using the laboratory-risk-score** | **p value for paired t-test between chronological age and PHA using the non-laboratory-risk-score** |
| --- | --- | --- | --- | --- | --- | --- | --- |
| Men | 26,213 | 46.4 | 53.0 | 54.7 | <0.001 | <0.001 | <0.001 |
| Women | 39,805 | 45.9 | 50.1 | 48.9 | <0.001 | <0.001 | <0.001 |

This analysis was restricted for population with measured high-density lipoprotein (HDL) cholesterol.

**Supplementary Table 3. Protocol for blood pressure, glucose, and total cholesterol measurements by country.**

| **Country** | **Year** | **Blood pressure measurement** | | | | **Glucose measurement** | | | | **Total cholesterol measurement** | |
| --- | --- | --- | --- | --- | --- | --- | --- | --- | --- | --- | --- |
|  |  | **Measurement device** | **Cuff sizes** | **Number of measurements** | **Time between measurements** | **Measurement device** | **Fasting time** | **Results expressed as plasma or whole blood?** | **Post Hoc adjustmentt** | **Measurement device** | **Fasting time** |
| Afghanistan | 2018 | Sphygmomanometer (model not specified) | Not reported | 3 | 3 minutes | POC capillary testing (Cardiocheck PA) | Not reported | Plasma | None | POC capillary testing (Cardiocheck PA) | Not reported |
| Algeria | 2017 | Digital upper arm meter (model not specified) | Not reported | 3 | Not reported | Not reported | Not reported | Not reported | None | Not reported | Not reported |
| Armenia | 2016 | Digital upper arm meter (model not specified) | Not reported | 3 | Not reported | Not reported | Not reported | Not reported | None | Not reported | Not reported |
| Azerbaijan | 2017 | Digital upper arm meter (Riester Ri-Champion Automatic Digital Monitor- 1715) | 3: small, medium, large | 3 | 10 minutes | Not reported | Not reported | Not reported | None | Not reported | Not reported |
| Bangladesh | 2018 | Digital upper arm meter (BOSO–Medicus Control with universal cuff) | Universal | 3 | 3 minutes | Laboratory (method not reported) | 12 hours | Plasma | None | Laboratory (method not reported) | 12 hours |
| Belarus | 2017 | Digital upper arm meter (Boso-Medicus Uno with universal cuff) | Universal | 3 | 3 minutes | POC capillary testing (Cardiocheck PA) | Not reported | Plasma | None | POC capillary testing (Cardiocheck PA) | Not reported |
| Benin | 2015 | Digital upper arm meter (Boso-Medicus Uno) | Not reported | 3 | 3 minutes | POC capillary testing (Cardiocheck PA) | Not reported | Plasma | None | POC capillary testing (Cardiocheck PA) | Not reported |
| Bhutan | 2019 | Omron digital upper arm meter (model not specified) | Universal | 3 | 5 minutes | POC capillary testing (Cardiocheck PA) | 12 hours | Plasma | None | POC capillary testing (Cardiocheck PA) | 12 hours |
| Botswana | 2014 | Digital upper arm meter (model not specified) | 3: small, medium, large | 3 | Not reported | Not reported | Not reported | Not reported | None | Not reported | Not reported |
| Brunei Darussalam | 2016 | Not reported | Not reported | 3 | Not reported | Not reported | Not reported | Not reported | None | Not reported | Not reported |
| Ecuador | 2018 | Digital upper arm meter (model not specified) | Universal | 3 | Not reported | POC capillary testing (Cardiocheck PA) | Not reported | Plasma | None | POC capillary testing (Cardiocheck PA) | Not reported |
| Eswatini | 2014 | Digital upper arm meter (Boso Medicus Uno) | 3: small, medium, large | 3 | 3-5 minutes | POC capillary testing (Cardiocheck PA) | Not reported | Plasma | None | POC capillary testing (Cardiocheck PA) | Not reported |
| Ethiopia | 2015 | Digital upper arm meter (model not specified) | Universal | 3 | Not reported | POC capillary testing (Cardiocheck PA) | 8 hours | Plasma | None | POC capillary testing (Cardiocheck PA) | 8 hours |
| Georgia | 2016 | Digital upper arm meter (Boso Medicus Uno) | 3: small, medium, large | 3 | 3 minutes | POC capillary testing (MultiCare-in) | Not reported | Plasma | None | POC capillary testing (MultiCare-in) | Not reported |
| Guyana | 2016 | Omron digital upper arm meter (model not specified) | Not reported | 3 | 3 minutes | POC capillary testing (Accutrend Plus) | 8 hours | Whole blood | Multiplied by 1.11 | Laboratory | 8 hours |
| Iraq | 2015 | Mercury sphygmomanometer (model not specified) | Not reported | 2 with a third measurement if the first two differed by >10 mmHg | Not reported | Laboratory (Glucose Oxidase method) | 10-14 hours | Plasma | None | Laboratory (Cholesterol Oxidase method) | 10-14 hours |
| Jordan | 2019 | Digital upper arm meter (model not specified) | Not reported | 3 | 3 minutes | POC capillary testing (Cardiocheck PA) | Not reported | Plasma | None | POC capillary testing (Cardiocheck PA) | Not reported |
| Kenya | 2015 | Omron M2 Digital Monitor | Not reported | 3 | 3-5 minutes | POC capillary testing (Cardiocheck PA) | 12 hours | Plasma | None | POC capillary testing (Cardiocheck PA) | 12 hours |
| Kiribati | 2016 | Omron M4 digital upper arm meter | Not reported | 3 | 2-3 minutes | POC capillary testing (Cardiocheck PA) | 8 hours | Plasma | None | POC capillary testing (Cardiocheck PA) | 8 hours |
| Kuwait | 2014 | Mercury sphygmomanometer (model not specified) | Universal | 3 | 3 minutes | Laboratory (hexokinase enzymatic method) | 12 hours | Plasma | None | Laboratory (enzymatic method) | 12 hours |
| Kyrgyzstan | 2013 | Digital upper arm meter (model not specified) | Not reported | 3 | Not reported | Not reported | Not reported | Not reported | None | Not reported | Not reported |
| Lebanon | 2017 | Manual mercury sphygmomanometer | Not reported | 3 | 5 minutes | Laboratory | Not reported | Plasma | None | Laboratory | Not reported |
| Malawi | 2017 | Omron M4-I digital upper arm meter (model not specified) | Not reported | 3 | 3-5 minutes | POC capillary testing (Cardiocheck PA) | Not reported | Plasma | None | POC capillary testing (Cardiocheck PA) | Not reported |
| Mongolia | 2019 | Digital upper arm meter Omron M5 Digital Monitor) | Not reported | 3 | 3 minutes | POC capillary testing (Prima home test) | Not reported | Plasma | None | POC capillary testing (Prima home test) | Not reported |
| Morocco | 2017 | Digital upper arm meter (Spengler® ES 60) | Not reported | 3 | "A few minutes" (exact time not reported) | POC capillary testing (Cardiocheck PA) | Not reported | Plasma | None | POC capillary testing (Cardiocheck PA) | Not reported |
| Myanmar | 2014 | Digital upper arm meter (Boso Medicus with universal cuff size) | Universal | 3 | 3 minutes | POC capillary testing (SD LipidoCare Analyzer) | Not reported | Plasma | None | POC capillary testing (SD LipidoCare Analyzer) | Not reported |
| Nauru | 2016 | Digital upper arm meter (model not specified) | Not reported | 3 | Not reported | POC capillary testing (Cardiocheck PA) | Not reported | Plasma | None | Not reported | Not reported |
| Nepal | 2019 | Omron digital upper arm meter with universal cuff size | Universal | 3 | 3 minutes | POC capillary testing (Cardiocheck PA) | Not reported | Plasma | None | POC capillary testing (Cardiocheck PA) | Not reported |
| Republic of Moldova | 2013 | Digital upper arm meter (Boso Medicus with universal cuff size) | Universal | 3 | 3 minutes | POC capillary testing (Cardiocheck PA) | Not reported | Plasma | None | POC capillary testing (Cardiocheck PA) | Not reported |
| Sao Tome and Principe | 2019 | Not reported | Not reported | 3 | Not reported | POC capillary testing (NovaPro) | Not reported | Not reported | None | POC capillary testing (Multicare devices) | Not reported |
| Solomon Islands | 2015 | Digital upper arm meter (model not specified) | Not reported | 3 | Not reported | POC capillary testing (Cardiocheck PA) | Not reported | Plasma | None | POC capillary testing (Cardiocheck PA) | Not reported |
| Sri Lanka | 2015 | Digital upper arm meter (model not specified) | 3: small, medium, large | 3 | Not reported | POC capillary testing (Cardiocheck PA) | Not reported | Plasma | None | POC capillary testing (Cardiocheck PA) | Not reported |
| Sudan | 2016 | Digital upper arm meter (Boso-Medicos Uno) | Not reported | 3 | 3 minutes | POC capillary testing (Cardiocheck PA) | >8 hours | Plasma | None | POC capillary testing (Cardiocheck PA) | >8 hours |
| Tajikistan | 2017 | Digital upper arm meter (model not specified) | Not reported | 3 | 5 minutes | POC capillary testing (Cardiocheck PA) | Not reported | Plasma | None | POC capillary testing (Cardiocheck PA) | Not reported |
| Timor-Leste | 2014 | Omron digital upper arm meter (model not specified) | Not reported | 3 | 2 minutes | POC capillary testing (Cardiocheck PA) | Not reported | Plasma | None | POC capillary testing (Cardiocheck PA) | Not reported |
| Tokelau | 2014 | Digital upper arm meter (model not specified) | Not reported | 3 | Not reported | POC capillary testing (Accutrend Glucose) | 9 hours | Whole blood | Multiplied by 1.11 | POC capillary testing (Accutrend GCT) | 9 hours |
| Turkmenistan | 2018 | Omron digital upper arm meter (model not specified) | Not reported | 3 | 3 minutes | POC capillary testing (Cardiocheck PA) | Not reported | Plasma | None | POC capillary testing (Cardiocheck PA) | Not reported |
| Tuvalu | 2015 | Digital upper arm meter (model not specified) | 3: small, medium, large | 3 | Not reported | POC capillary testing (Accu-chek Performa) | Not reported | Plasma | None | POC capillary testing (Accutrend Plus) | Not reported |
| Uganda | 2014 | Digital upper arm meter (Boso Medicus Uno) | 3: small, medium, large | 3 | 3-5 minutes | POC capillary testing (Cardiocheck PA) | 8 hours | Plasma | None | POC capillary testing (Cardiocheck PA) | 8 hours |
| Vietnam | 2015 | Digital upper arm meter (BOSO device) | Not reported | 3 | Not reported | POC capillary testing (Cardiocheck PA) | Not reported | Plasma | None | POC capillary testing (Cardiocheck PA) | Not reported |
| Zambia | 2017 | Digital upper arm meter (model not specified) | Not reported | 3 | 3-5 minutes | POC capillary testing (Cardiocheck PA) | >10 hours | Plasma | None | POC capillary testing (Cardiocheck PA) | >10 hours |

POC: Point of care (i.e., portable device)

Note1: all information was extracted, as reported, from the official reports available with the datasets in the online data repository.

Note2: Post hoc adjustment to convert from whole blood to plasma equivalents.

|  |  |  |  |  |  |  |  |  |  |  |  |
| --- | --- | --- | --- | --- | --- | --- | --- | --- | --- | --- | --- |

**
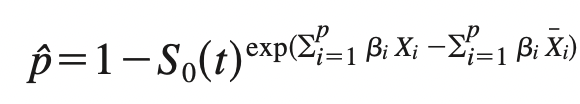
****Supplementary Figure 2. Formula to estimate cardiovascular disease (CVD) risk using the Framingham risk score.**

where S0(t) is baseline survival at follow-up time t (e.g., 10 years), βi is the estimated regression coefficient (log hazard ratio), Xi is the log-transformed the value of the *ith* risk factor, X̄i is the corresponding mean, and p denotes the number of risk factors.

**Supplementary Figure 3. Distribution of predicted heart age (PHA) groups by chronological age groups in Africa, by sex.**

# **Supplementary Figure 4. Distribution of predicted heart age (PHA) groups by chronological age groups in Americas, by sex**

# **Supplementary Figure 5. Distribution of predicted heart age (PHA) groups by chronological age groups in South-East Asia, by sex**

# **Supplementary Figure 6. Distribution of predicted heart age (PHA) groups by chronological age groups in Europe, by sex.**

# **Supplementary Figure 7. Distribution of predicted heart age (PHA) groups by chronological age groups in Eastern Mediterranean, by sex.**

# **Supplementary Figure 8. Distribution of predicted heart age (PHA) groups by chronological age groups in Western Pacific, by sex.**

# **Supplementary Figure 9. Proportion (%) of very high predicted heart age (PHA) by survey year.**

# **Supplementary Figure 10. Proportion (%) of low, equal, high, and very high predicted heart age (PHA) by country and quintile of absolute cardiovascular disease risk, stratified by region.**

# **Supplementary Figure 11. Proportion (%) of people with very high predicted heart age (PHA) who are eligible/not eligible for antihypertensive treatment based on guideline recommendations.**

# **Supplementary Figure 12. Proportion (%) of people with predicted heart age (PHA) exceeding 10 years who are eligible/not eligible for antihypertensive treatment based on guideline recommendations.**

# **Supplementary Figure 13. Proportion (%) of people with predicted heart age (PHA) exceeding 20 years who are eligible/not eligible for antihypertensive treatment based on guideline recommendations.**

# **Supplementary Figure 14. Proportion (%) of people with very high predicted heart age (PHA) who are eligible/not eligible for lipid-lowering treatment based on guideline recommendations.**

# **Supplementary Figure 15. Proportion (%) of people with predicted heart age (PHA) exceeding 10 years who are eligible/not eligible for lipid-lowering treatment based on guideline recommendations.**

# **Supplementary Figure 16. Proportion (%) of people with predicted heart age (PHA) exceeding 20 years who are eligible/not eligible for lipid-lowering treatment based on guideline recommendations.**
